# The Risk of Herpes Zoster Events in Patients with Spondyloarthritis and the Effect of BNT162b2 mRNA COVID-19 Vaccine

**DOI:** 10.3390/vaccines12010085

**Published:** 2024-01-15

**Authors:** Tal Gazitt, Noa Hayat, Nili Stein, Amir Haddad, Ilan Feldhamer, Arnon Dov Cohen, Walid Saliba, Devy Zisman

**Affiliations:** 1 Rheumatology Unit, Carmel Medical Center, Haifa 3436212, Israel; amirha3@clalit.org.il (A.H.); devyzi@clalit.org.il (D.Z.); 2 Division of Rheumatology, University of Washington Medical Center, Seattle, WA 98195-6428, USA; 3 Department of Internal Medicine, Carmel Medical Center, Haifa 3436212, Israel; noahay@clalit.org.il; 4 Department of Community Medicine and Epidemiology, Carmel Medical Center, Haifa 3436212, Israelsaliba_wa@clalit.org.il (W.S.); 5 Chief Physician’s Office, Central Headquarters, Clalit Health Services, Tel Aviv 67754, Israel; 6 Siaal Research Center for Family Medicine and Primary Care, Faculty of Health Sciences, Ben-Gurion University of the Negev, Beer-Sheva 84105, Israel; 7 The Ruth and Bruce Rappaport Faculty of Medicine, Technion, Haifa 31096, Israel

**Keywords:** spondyloarthritis, ankylosing spondylitis, psoriatic arthritis, mRNA vaccine, COVID-19, herpes zoster

## Abstract

The data on the risk of herpes zoster (HZ) in spondyloarthropathy (SpA) patients are sparse, especially regarding its association with the novel mRNA COVID-19 vaccines and immunosuppressants. We aimed to evaluate whether SpA diagnosis and/or immunosuppressant use affect HZ risk and the influence of mRNA COVID-19 vaccination. We assessed the association between SpA (psoriatic arthritis (PsA) and ankylosing spondylitis (AS)) diagnoses and HZ in a large population database with patients matched by age and sex to controls. We also assessed the association between the COVID-19 vaccine and new-onset HZ using two nested case–control studies, identifying all new HZ cases diagnosed from 1 January–31 December 2021 within the SpA and general population cohorts, matched randomly by sex, age and HZ index date to controls without HZ. Exposure to mRNA COVID-19 vaccination was ascertained in the 6 weeks prior to the index date both in cases and controls. In our results, the incidence rate of HZ was higher in PsA patients vs. the general population, at 1.03 vs. 0.64 per 100 person-years, respectively (adjusted HR = 1.55; 95%CI, 1.19–2.02). Within the SpA group, Jak-I treatment was associated with a higher risk of developing new-onset HZ (adjusted OR = 3.79; 1.15–12.5). Multivariable conditional logistic regression models we used showed no association between COVID-19 vaccination and new-onset HZ among the SpA patients (OR = 1.46; 0.68–3.14).

## 1. Introduction

Varicella zoster virus (VZV) can manifest initially as chickenpox. Following infection, the virus remains dormant in the sensory ganglia and holds the potential to become reactivated and cause herpes zoster (HZ) even years later [1]. Several risk factors are known to cause HZ reactivation, such as older age, immunodeficiency (including malignancies, chemotherapy, high-dose glucocorticosteroid (GC) treatment, human immunodeficiency virus (HIV) infection, and bone marrow or solid organ transplantation), family history of HZ, physical trauma, psychological stress, female sex, and various comorbidities, including diabetes mellitus (DM), cardiovascular disease (CVD), hypertension (HTN), chronic obstructive pulmonary disease (COPD), asthma, chronic kidney disease (CKD), depression, inflammatory bowel disease (IBD), and autoimmune inflammatory rheumatic diseases (AIIRD), such as rheumatoid arthritis (RA) and systemic lupus erythematosus (SLE) [2,3,4,5]. Importantly, vaccinations, including the recent mRNA vaccines developed against SARS-CoV2, have also been associated with increase in HZ risk [6,7]. Notably, even a few cases of HZ found in patients who previously received the live-attenuated Zostravax^®^ (Merck & Co., Rahway, NY, USA) vaccination against VZV were found to be caused by reactivation of the virus found in the vaccine, unrelated to community-type varicella [8].

To date, several studies have suggested that there is no increase in risk of HZ among SpA patients relative to comparator populations. For instance, in the case of AS, a study by Wong et al. showed no increase in the incidence of HZ in AS relative to RA (stratified HR = 0.97; 0.58–1.61) [9], while a Taiwanese study by Wang et al. showed no difference in the incidence rate of HZ between AS patients and non-AS controls (adjusted HR = 1.07; 0.84–1.37), with increasing age and cancer serving as significant risk factors for incidental HZ [10]. In the case of PsA, recent studies mostly discuss the risk of HZ in relation to baseline immunosuppressive treatments [11,12], but a previous study also relying on the CHS database demonstrated that the risk of HZ in an untreated PsA cohort without previous Zostravax^®^ vaccination is 9.21/1000 patient-years [13], which is somewhat higher than that in the general population, which is around 7–10/1000 after 50 years of age [14].

Several studies suggest an increase in the risk of HZ events in patients treated with biologic disease modifying anti-rheumatic drugs (bDMARDs), but the association between HZ events and the use of bDMARDs remains unclear [15,16,17,18,19,20,21,22,23]. While most of the studies on this subject thus far included only RA patients, some studies also included patients with psoriasis (PsO) [24] and psoriatic arthritis (PsA) [12,13,25]. Of these studies, some implicate anti-TNF-α therapy in augmenting HZ risk [11,12], while others find no association between HZ events and therapy with bDMARDs alone in these patients [13,24,25].

Overall, the data on the risk of HZ in patients with SpA are sparse in general and in particular in terms of its association with the novel mRNA vaccines and DMARD use. Therefore, we aimed to evaluate whether an underlying SpA diagnosis (PsA vs. AS), treatment with immunosuppressive medications or the BNT162b2 mRNA COVID-19 vaccine is associated with an increase in the risk of HZ occurrence in SpA patients vs. the general population.

## 2. Methods

### 2.1. Source of Data

This retrospective cohort study is based on the database of Clalit Health Services (CHS), the largest health care provider in Israel. This database includes information on approximately 4.7 million members, constituting ~52% of the population in Israel. This cohort and the validity of algorithms used previously by our group to identify patients with PsA and AS are described in detail elsewhere [26,27,28].

This database study was approved by the Institutional Review Board of Carmel Medical Center (CMC-0014-14). The requirement for individual patient consent forms was waived due to the retrospective, observational nature of the study.

### 2.2. Study Cohort

To assess the association between SpA diagnosis and HZ, we conducted the following retrospective cohort study: All adult members of CHS aged 18 years or older with a previous diagnosis of PsA or AS prior to the cohort entry date of 1 January 2021 were included in the study. These individuals were frequency-matched by year of birth ± 1 years as well as by sex to patients without SpA diagnosis at a 1:10 ratio. All of these cohort members were followed until a new diagnosis of HZ, death, or end of follow-up on 31 December 2021, whichever came first.

To assess the association between the BNT162b2 mRNA COVID-19 vaccine and new-onset HZ, we conducted two separate nested case–control studies involving (1) an SPA cohort consisting of CHS members aged 18 years or older as of 1 January 2022 with a previous diagnosis of PsA or AS prior to the cohort entry date of 1 January 2021; (2) a control group consisting of CHS members aged 18 years or older as of 1 January 2022 without previous SpA diagnosis. Within the SpA and control groups, we identified all new HZ cases diagnosed during follow-up until 31 December 2021. For each HZ case, with the HZ date serving as the index date, we randomly selected 10 controls who did not incur HZ within the SpA group or general population group based on age, sex, and index date using risk set sampling. Exposure to mRNA COVID-19 vaccination was ascertained in the 6 weeks (42 days) prior to the index date in both cases and controls. The analysis was conducted separately for the SpA group and for the general population group.

### 2.3. Data Collected

For each individual enrolled in the study, the following data were retrieved from the CHS database: demographic variables, smoking status, socio-economic status (SES), body mass index (BMI), presence of selected chronic comorbidities, and data on medication use by SpA patients, including use of glucocorticosteroids (GCs) and DMARDs, which were divided into the following groups: (1) conventional DMARDs (cDMARDs, including methotrexate, sulfasalazine, leflunomide, cyclosporine A, azathioprine, and hydroxycholoroquine and the small-molecule inhibitor apremilast), (2) biologic DMARDs (bDMARDs, including the anti-TNF-α agents infliximab, adalimumab, golimumab, certolizumab pegol, and etanercept; the anti-IL-17 agents secukinumab and ixekizumab; the anti-IL-12/23 inhibitor ustekinumab; the anti-IL-23 inhibitors guselkumab and risankizumab); and (3) targeted-synthetic DMARDs (tsDMARDs, including the janus kinase inhibitors (Jak-I) tofacitinib and upadacitinib), as well as each individual’s HZ vaccination status using the live-attenuated HZ vaccine available in Israel during the study period (Zostravax^®^, Merck & Co., Rahway, NY, USA).

### 2.4. Study Outcome

The study outcome was defined as the occurrence of an HZ event. The diagnosis of HZ was based on ICD-9 codes (053.0–053.9) with concurrent issuance of acyclovir by patient within ±14 days of the diagnostic code being assigned by the provider.

### 2.5. Statistical Methods

Continuous variables were summarized with means and standard deviations (SDs), and categorical variables were summarized with counts and proportions.

To estimate the crude and adjusted hazard ratio (HR) for the association between underlying SpA diagnosis and HZ occurrence, we used Cox proportional hazard models. To estimate the odds ratio (OR) for the association between the mRNA COVID-19 vaccine and new-onset HZ and the odds ratio for the association between DMARD use and HZ events, we used conditional logistic regression. The multivariable models were adjusted for age to account for residual confounding after matching, prior history of COVID-19, SES, population sector, tobacco use, BMI, psoriasis, cancer, diabetes, ischemic heart disease (IHD), chronic heart failure (CHF), cerebrovascular accident/transient ischemic attack (CVA/TIA), CRF, HTN, COPD, prior HZ vaccine, and prior HZ.

Statistical analyses were performed using SAS version 9.4 (SAS Institute Inc., Cary, NC, USA) and SPSS version 28 (IBM Corp. Released 2022. IBM SPSS Statistics for Windows, version 28.0, 2022, Armonk, NY, USA). For all analyses, *p* < 0.05 (for the 2-tailed tests) was considered statistically significant.

## 3. Results

Our study population included 6274 PsA patients, 2545 AS patients, and their matched controls, with 62,740 controls matched to the PsA group and 25,450 to the AS group (Table 1). In the PsA group, males constituted 45.8% of patients, and the average age was 58.4 ± 15.2 years. In the AS group, males constituted 52.3% of patients, and the average age was 49.4 ± 14.5 years.

Demographic differences between PsA patients and AS patients and their respective comparative groups from the general population included Jewish ethnicity, higher tobacco use, higher BMI, lower SES, and a higher prevalence of CVD-related comorbidities as well as CRF and COPD (Table 1). In addition, there was a higher prevalence of prior HZ vaccination and prior HZ occurrence in the PsA and AS subgroups relative to the general population. When comparing the PsA and AS subgroups, PsA patients were more likely to be female and older on average than AS patients.

Among the PsA patients, 64 developed new-onset HZ during 6182 person-years of follow-up (incidence rate 1.03 per 100 person-years) compared with 398 new HZ cases in the comparative group during 61,975 person-years (incidence rate 0.64 per 100 person-years) (adjusted HR = 1.55; 1.19–2.02). Among the AS patients, 18 developed new-onset HZ during 2522 person-years of follow-up (incidence rate 0.71 per 100 person-years) compared with 127 new HZ cases in the comparative group during 25,247 person-years (incidence rate 0.50 per 100 person-years) (adjusted HR = 1.38; 0.84–2.27) (Table 2).

In our nested case–control study designed to examine the association between mRNA COVID-19 vaccination and new-onset HZ in the CHS database, a total of 3,114,449 individuals from the general population without underlying SpA and 8819 SpA patients constituted the starting cohort. From this initial cohort, 13,335 individuals from the general population and 82 patients within the SpA group developed new-onset HZ and were matched with 133,350 and 820 controls without HZ, respectively (Table 3).

Within the SpA group, 24/82 (29.3%) HZ cases received the BNT162b2 mRNA COVID-19 vaccine in the prior 6 weeks compared to 211/820 (25.7%) in their matched controls. Among the general population, 3213/13,335 (24.1%) HZ cases received the BNT162b2 mRNA COVID-19 vaccine in the prior 6 weeks compared to 30,601/133,350 (22.9%) in their matched controls. Within the SpA group, there was no association between bDMARDs and HZ events (Anti-TNF-α agents, *p* = 0.179; anti-IL-17 agents, *p* = 0.615; anti-IL-23 agents, *p* = 0.676). Similarly, cDMARDs were not associated with increased HZ incidence (methotrexate, *p* = 0.511; sulfasalazine, *p* = 0.325; leflunomide, *p* = 0.299; apremilast, *p* = 0.465; hydroxychloroquine, *p* = 0.119). Jak-I treatment, however, was found to be associated with a higher risk of developing new-onset HZ compared to SpA patients with no treatment or other treatments (crude OR = 3.89 (1.18–12.8); adjusted OR = 3.79 (1.15–12.5)) (Table 3).

In multivariable conditional logistic regression analysis, the adjusted-OR associated with mRNA COVID-19 vaccine use was 1.46 (0.68–3.14) in the SpA group and 1.08 (1.02–1.14) in the general population (Table 4). The *p* for interaction was 0.450.

## 4. Discussion

To date, the data on the risk of HZ events in SpA patients have been sparse. In our study, we found that there is an increase in risk of HZ in PsA patients relative to the general population, which is not seen in AS patients. While we only observed a very small number of HZ events in the AS subgroup, which may have hindered the detection of an added HZ risk in this subgroup relative to the general population, our results on the risk of HZ in AS patients are in line with a Taiwanese study by Wang et al. [10], which, after adjusting for age, sex, and systemic medications, found the hazard ratio (HR) for HZ events in AS patients to be 1.07 (0.84–1.37) compared to non-AS controls. Additionally, similar to our study results, patients with psoriasis and PsA were found to have higher rates of HZ (13.3 and 15.9 cases/1000 person-years, respectively) compared with healthy controls (8.5 cases/1000 person-years) when adjusted for age, sex, and systemic medications [29,30].

Our results highlighting HZ risk particularly in PsA patients are of interest, as they suggest that the difference in HZ risk between PsA and AS patients may stem from underlying disease-related factors or treatments regimens. One possible reason for this difference in HZ risk between PsA and AS patients may be related to underlying demographic factors typical for PsA vs. AS, in that PsA patients are typically older patients and more often female than AS patients [31], as was the case in our study. Accordingly, older age and female sex have been shown to be associated with higher HZ risk [5], as also seen in the PsA and AS cohorts in our study, though not in the general population.

Moreover, the high CVD-related risk burden attributed to PsA, which is thought to exist even independently of the traditional CVD-related comorbidities also known to be associated with PsA [32], may also affect HZ risk in PsA patients relative to AS patients. In this regard, HZ itself has been shown to be associated with CVD-related risk factors, both in the general population [3,4,5] as well as in AIIRD patients [2]. Indeed, among the comorbidities we studied, we found a higher risk of HZ events in individuals with traditional CVD-related risk factors, both in the general population and in the separate analyses on PsA and AS patients, although this risk was not captured in the combined analysis of the entire SpA group in our nested case–control study.

When it comes to vaccine-related HZ risk, only a very small percentage of individuals in the general population and in the SpA group received the Zostravax^®^ vaccine prior to the study entrance date (notably, this live-virus vaccination is contraindicated in patients on immunosuppressive medications); therefore, there is low likelihood that many individuals experienced Zostravax^®^-related HZ reactivation. Moreover, given the small percentage of vaccinated individuals, no definitive conclusions can be drawn regarding the seroprotectivity of Zostravax^®^ in our study cohort.

To date, there have been several reports on HZ events following non-mRNA COVID-19 vaccinations in the general population. For instance, an early case series published in 1999 [7] documented HZ events following vaccines against hepatitis A virus, influenza virus, and rabies. A recent study [33] utilizing Taiwan’s National Health Insurance Research Database between 2015 and 2017 found a slight increase in risk of HZ (incident risk ratio (IRR) = 1.11 (1.02–1.20)) in people receiving the influenza vaccine in the first 1–15 days after vaccination. The pioneering use of mRNA vaccines in clinical practice has raised concern for a potentially increased risk for HZ reactivation because HZ reactivation occurs in the setting of a decline in cell-mediated immunity [34,35], and mRNA COVID-19 vaccines elicit the activation of CD4+ and CD8+ T-cells and a TH1-mediated immune response, which could potentially interfere with the T-cell-mediated immunity directed against VZV [36,37]. Since the advent of mRNA COVID-19 vaccinations, several publications have described HZ events occurring in patients within a few days after receiving a dose of an mRNA COVID-19 vaccine [38,39]. A large study using the worldwide pharmacovigilance database VigiBase [40] and disproportionality analyses (case/non-case statistical approach) to assess the relative risk of HZ reporting in mRNA COVID-19 vs. influenza vaccine recipients found that mRNA COVID-19 vaccines were associated with a significantly higher reporting of HZ (reporting odds ratio = 1.9; CI = 1.8–2.1), with most events reported as mild and occurring in individuals over 40 years of age.

Reassuringly, although AIIRD patients are known to be at increased risk of infections [41,42,43] and specifically of HZ reactivation [5,44], we did not find an increase in the risk of HZ reactivation following mRNA vaccination in SpA patients relative to the general population. This finding is particularly noteworthy given that the AIIRD patient population has been aggressively immunized against COVID-19 due to the concern of potentially more severe outcomes of this infection in immunosuppressed individuals and particularly in those with CVD-related comorbidities, as found in PsA patients [32,45,46].

Of note, in a separate study also conducted on the CHS database by Barda et al. [39] and aimed at evaluating adverse events occurring within 42 days of BNT162b2 mRNA COVID-19 vaccination, the risk ratio for HZ in the general population of individuals above 16 years of age was 1.43 ((1.20–1.73); risk difference, 15.8 events per 100,000 persons; (8.2–24.2)). In our study, which was performed on the same CHS database, only a very mild increase in HZ risk following mRNA vaccination was observed within the general population group, which is not considered clinically significant. The difference in HZ risk found within the general population in our study vs. Barda et al.’s study may stem from differences in the baseline definition of HZ events in the two studies in that we required a concurrent prescription of acyclovir in addition to the recorded diagnostic code corresponding to HZ for an event to be classified as an HZ event. This requirement increases the specificity of the diagnosis, as was done in a nation-wide study on HZ incidence in Denmark [47], but concomitantly lowers the sensitivity of detection of mild cases of HZ. Thus, this difference in the case definition of HZ may have led to the higher number of HZ cases found in Barda’s study relative to ours.

Thus far, only a few case series have analyzed the association between immunosuppressive medications and HZ occurrence in AIIRD patients receiving mRNA COVID-19 vaccines [48,49]. For instance, in a large, multi-center prospective observational study [50] on the safety of the BNT162b2 mRNA vaccine in AIIRD patients on a wide variety of cDMARDs and bDMARDs vs. controls, the incidence of HZ events (all non-disseminated) following mRNA COVID-19 vaccination was 6/670 (0.90%), with 5 cases occurring after the first vaccine dose and in 1 patient following the second vaccine dose. Another study from a large tertiary rheumatology referral center in Taiwan [36] on HZ events occurring in AIIRD patients following mRNA-1273 vaccination demonstrated that up to 6.2% of AIIRD patients developed HZ post-vaccination. In this study, most AIIRD patients were on cDMARDs or low-dose GC, and none were on bDMARDs or tsDMARDs.

In our study, the risk of HZ events in SpA patients was not increased in patients who were treated with cDMARDs, TNF-α inhibitors, and other biologics but was increased with the tsDMARDs, specifically Jak-I. Our data are inconsistent with several studies and case reports describing a possible association between HZ and various drug therapies for rheumatic diseases [12,36,48,49,50,51]. Several possible explanations exist for these differences. First, each study focused on a different study population. For instance, in the study by Katsikas et al. [51], most of the patients had RA, while the studies by Lee et al. [36] and Furer et al. [50] included numerous rheumatic diseases, including RA, SpA, SLE, vasculitis, and inflammatory myositis, while ours focused on SpA patients. Second, some DMARDs which are known to be associated with HZ reactivation, such as rituximab, mycophenolate mofetil (MMF), and GC [36,50], are not utilized in the treatment arsenal of SpA and therefore were not included in our study. However, Jak-I, which is used in the treatment regimen of PsA and known to be associated with HZ risk [11,12,52], was represented in our study and indeed found to be associated with an increased risk of HZ reactivation.

Possible limitations in our study include the relatively small number of HZ events captured in the CHS database, particularly in the AS subgroup. Additional general limitations of administrative database research include the lack of data on PsA and AS disease activity, as well as any data on the severity of HZ events, the potential presence of unmeasured confounders, and the potential misclassification of cases/controls and HZ events.

Despite the aforementioned limitations, our study on HZ risk in SpA patients was founded on a database of 4.7 million individuals featuring data on the long-term follow-up of PsA and AS patients in real life and the comparison of this SpA population to a large, matched control population. It is also of importance that it is one of the few studies examining the incidence of HZ following the novel mRNA vaccines in the immunosuppressed SpA patient population and is of particular relevance especially in light of the recent rise in SARS-CoV2 cases (World Health Organization (WHO) Coronavirus (COVID-19) Dashboard, https://COVID19.who.int last accessed 12 January 2024).

## 5. Conclusions

In our study, we found an inherent risk in HZ occurrence specifically among PsA patients relative to AS patients and the general population. However, we found no increase in HZ events in SpA patients following BNT162b2 mRNA vaccine doses relative to general population controls. Overall, these study results support the safety of mRNA COVID-19 vaccination in AIIRD patients.

## Figures and Tables

**Table 1:** Baseline characteristics of the study population for the association between SpA diagnosis and a new-onset HZ.

	General Population	PsA	*p*-Value	General Population	AS	*p*-Value *
**Number of patients**	62,740	6274		25,450	2545	
**Age** (mean ± SD)	58.4 ± 15.2	58.4 ± 15.2	0.951	49.4 ± 14.5	49.4 ± 14.5	0.985 ^a^ <0.001 ^b^
**Male** N (%)	28,740 (45.8)	2874 (45.8)		13,310 (52.3)	1331 (52.3)	>0.99 ^a^ <0.001 ^b^
**Jewish Ethnicity N (%)**	50,526 (80.5)	5385 (85.8)	<0.001	19,575 (76.9)	2065 (81.1)	<0.001 ^a^ <0.001 ^b^
**Socio-economic status**						
Low	22,728 (36.2)	1939 (30.9)	<0.001	10,055 (39.5)	914 (35.9)	<0.001 ^a^ <0.001 ^b^
Medium	25,990 (41.4)	2666 (42.5)		9777 (38.4)	1090 (42.8)	
High	13,888 (22.1)	1667 (26.6)		5579 (21.9)	540 (21.2)	
Missing	134 (0.2)	2 (0.03)		39 (0.2)	1 (0.04)	
**Tobacco use**	24,691 (39.4)	2820 (44.9)	<0.001	10,091 (39.7)	1246 (49.0)	<0.001 ^a^ <0.001 ^b^
**BMI**						
<25	19,594 (31.2)	1632 (26.0)	<0.001	8736 (34.3)	596 (32.9)	<0.001 ^a^ <0.001 ^b^
>=25 and <30	21,911 (34.9)	2281 (36.4)		8218 (32.3)	613 (33.8)	
>=30	16,975 (27.1)	2154 (34.3)		5728 (22.5)	517 (28.5)	
Missing	4260 (6.8)	207 (3.3)		2768 (10.9)	127 (5.0)	
**Psoriasis**	899 (1.4)	5222 (83.2)	<0.001	326 (1.3)	93 (3.7)	<0.001 ^a^ <0.0001 ^b^
**Cancer**	6540 (10.4)	744 (11.9)	<0.001	1631 (6.4)	207 (8.1)	0.002 ^a^ <0.001 ^b^
**Diabetes**	13,043 (20.8)	1671 (26.6)	<0.001	3367 (13.2)	395 (15.5)	0.001 ^a^ <0.001 ^b^
**IHD**	6804 (10.8)	898 (14.3)	<0.001	1630 (6.4)	185 (7.3)	0.091 <0.001 ^b^
**CVA/TIA**	3766 (6.0)	472 (7.5)	<0.001	856 (3.4)	96 (3.8)	0.278 ^a^ <0.001 ^b^
**CRF**	2492 (4.0)	324 (5.2)	<0.001	542 (2.1)	79 (3.1)	0.001 ^a^ <0.001 ^b^
**HTN**	19,820 (31.6)	2407 (38.4)	<0.001	4636 (18.2)	541 (21.3)	<0.001 ^a^ <0.001 ^b^
**COPD**	2193 (3.5)	340 (5.4)	<0.001	523 (2.1)	83 (3.3)	<0.001 ^a^ <0.001 ^b^
**CHF**	1674 (2.7)	213 (3.4)	<0.001	369 (1.4)	45 (1.8)	0.205 ^a^ <0.001 ^b^
**Prior HZ vaccine**	1306 (2.1)	275 (4.4)	<0.001	259 (1.0)	75 (2.9)	<0.001 ^a^ 0.002 ^b^
**Prior HZ**	3001 (4.8)	417 (6.6)	<0.001	806 (3.2)	119 (4.7)	<0.001 ^a^ <0.001 ^b^

* ^a^ General population vs. AS. ^b^ PsA vs. AS. Abbreviations: AS = ankylosing spondylitis, BMI = body mass index, CHF = Congestive Heart Failure, COPD = chronic obstructive pulmonary disease, CRF = chronic renal failure, CVA = cerebrovascular accident, HTN = hypertension, HZ = herpes zoster, IHD = ischemic heart disease, N = number, PsA = psoriatic arthritis, SD = standard deviation, TIA = transient ischemic attack.

**Table 2:** Crude and adjusted * hazard ratios (HRs) for the association between specific SpA diagnosis (PsA and AS) and herpes zoster (HZ) events.

Study Group	No. of Events	Follow-Up Duration (Person-Years)	Incidence Rate (95%CI) per 100 Person-Years of Follow-Up)	HR 95%CI	Adj HR 95%CI	*p*-Value
**PsA**						
**Controls**	398	61,975	0.64 (0.58–0.71)	Ref		
**PsA**	64	6182	1.03 (0.80–1.31)	1.61 (1.24–2.1)	1.55 (1.19–2.02)	0.001
**AS**						
**Controls**	127	25,246	0.50 (0.42–0.60)	Ref		
**AS**	18	2522	0.71 (0.42–1.1)	1.42 (0.87–2.32)	1.38 (0.84–2.27)	0.199

Abbreviations: Adj = adjusted, AS = ankylosing spondylitis, CI = confidence interval, HR = hazard ratio, PsA = psoriatic arthritis. * The multivariable models were adjusted for age to account for residual confounding after matching, prior history of COVID-19, SES, population sector, tobacco use, BMI, psoriasis, cancer, diabetes, IHD, CHF, CVA/TIA, CRF, HTN, COPD, prior HZ vaccine, and prior HZ.

**Table 3:** Baseline characteristics of the study population for the association between mRNA COVID-19 vaccine and new-onset HZ.

	General Population		*p*-Value	SpA		*p*-Value
	Cases N = 13,335	Controls N = 133,350		Cases N = 82	Controls N = 820	
**Age** (mean ± SD)	58.1 ± 18.1	57.8 ± 18.3	<0.001	62.7 ± 12.7	62.7 ± 12.6	0.988
**Male** N (%)	5513 (41.3)	55,130 (41.3)	N.A	25 (30.5)	250 (30.5)	N.A
Jewish Ethnicity N (%)	11,324 (84.9)	107,104 (80.3)	<0.001	75 (91.5)	720 (87.8)	0.320
**Socio-economic status**						
Low	4327 (32.4)	37,278 (39.5)	<0.001	27 (32.9)	219 (26.7)	0.456
Medium	5714 (42.8)	37,600 (39.9)		34 (41.5)	382 (46.6)	
High	3290 (24.7)	18,791 (19.9)		21 (25.6)	219 (26.7)	
Missing	4 (0.03)	328 (0.2)		0	3 (0.6)	
**Tobacco use**	3653 (38.7)	35,223 (37.4)	0.006	23 (43.4)	238 (44.9)	0.830
**BMI**						
<25	4697 (35.2)	46,580 (34.9)	<0.001	25 (30.5)	210 (25.6)	0.284
>=25 and <30	4811 (36.1)	46,073 (34.6)		33 (40.2)	303 (37.0)	
>=30	3525 (26.4)	35,271 (26.4		23 (28.0)	300 (36.6)	
Missing	302 (2.3)	5426 (4.1)		1 (1.2)	7 (0.9)	
**Psoriasis**	194 (1.5)	1906 (1.4)	0.950	52 (63.4)	540 (65.9)	0.578
**Cancer**	1810 (13.6)	14,712 (11.0)	<0.001	15 (18.3)	124 (15.1)	0.444
**Diabetes**	2868 (21.5)	27,862 (20.9)	0.074	21 (25.6)	256 (31.2)	0.273
**IHD**	1637 (12.3)	14,994 (11.2)	<0.001	9 (11.0)	116 (14.1)	0.391
**CVA/TIA**	869 (6.5)	8805 (6.6)	0.691	7 (8.5)	73 (8.9)	0.909
**CRF**	565 (4.2)	5942 (4.5)	0.223	4 (4.9)	39 (4.8)	0.960
**HTN**	4532 (34.0)	43,861 (32.9)	0.002	40 (48.8)	359 (43.8)	0.321
**COPD**	511 (3.8)	4806 (3.6)	0.173	4 (4.9)	63 (7.7)	0.355
**CHF**	447 (3.4)	4094 (3.1)	0.069	2 (2.4)	35 (4.3)	0.389
**GC**	1022 (7.7)	5166 (3.9)	<0.001	10 (12.2)	88 (10.7)	0.681
**Prior COVID-19**	943 (10.0)	9460 (10.0)	0.921	3 (5.7)	60 (11.3)	0.203
**Prior HZ vaccine**	228 (1.7)	2838 (2.1)	0.001	5 (6.1)	39 (4.8)	0.589
**Prior HZ**	1021 (7.7)	7088 (5.3)	<0.001	12 (14.6)	72 (8.8)	0.097
**bDMARDs**				29 (35.4)	254 (31.0)	0.411
**cDMARDs**				17 (20.7)	179 (21.8)	0.814
**tsDMARDs (Jak-I)**				4 (4.9)	11 (1.3)	0.026

Abbreviations: b/c/ts DMARD = biologic/conventional/targeted synthetic disease-modifying anti-rheumatic drugs, BMI = body mass index, CHF = Congestive Heart Failure, COPD = chronic obstructive pulmonary disease, CRF = chronic renal failure, CVA = cerebrovascular accident, GC = glucocorticosteroids, HTN = hypertension, HZ = herpes zoster, IHD = ischemic heart disease, N = number, N.A. = non-applicable, PCR = polymerase chain reaction, SD = standard deviation, SpA = spondyloarthropathy, TIA = transient ischemic attack.

**Table 4:** Univariable and multivariable * models for the association between COVID-19 vaccine and new-onset HZ.

	N	Exposure (COVID-19 Vaccine Prior 6 Weeks)	Crude OR (95%CI)		Adjusted OR (95%CI)	
**General population**						
**HZ**						
No	133,335	30,601 (22.9)	Reference		Reference	
Yes	13,335	3213 (24.1)	1.11 (1.05–1.17)	<0.001	1.08 (1.02–1.14)	0.011
**SpA population**						
**HZ**						
No	820	211 (25.7)	Reference		Reference	
Yes	82	24 (29.3)	1.49 (0.70–3.16)	0.304	1.46 (0.68–3.13)	0.334

Abbreviations: CI = confidence interval, HZ = herpes zoster, N = number, OR = odds ratio, SpA = spondyloarthropathy. * The multivariable models were adjusted for age to account for residual confounding after matching, prior history of COVID-19, SES, population sector, tobacco use, BMI, psoriasis, cancer, diabetes, IHD, CHF, CVA/TIA, CRF, HTN, COPD, prior HZ vaccine, and prior HZ.

## Data Availability

The data used and/or analyzed during the present study are available from the corresponding author on reasonable request.
